# Comparing the accuracy of two secondary food environment data sources in the UK across socio-economic and urban/rural divides

**DOI:** 10.1186/1476-072X-12-2

**Published:** 2013-01-17

**Authors:** Thomas Burgoine, Flo Harrison

**Affiliations:** 1 UKCRC Centre for Diet and Activity Research (CEDAR), Institute of Public Health, Box 296, Forvie Site, Robinson Way, University of Cambridge, Cambridge, CB2 0SR, UK; 2 UKCRC Centre for Diet and Activity Research (CEDAR), Norwich Medical School, University of East Anglia, Norwich, NR4 7TJ, UK

**Keywords:** Food environment, Secondary data, Data completeness, Geographic information systems

## Abstract

**Background:**

Interest in the role of food environments in shaping food consumption behaviours has grown in recent years. However, commonly used secondary food environment data sources have not yet been fully evaluated for completeness and systematic biases. This paper assessed the accuracy of UK Points of Interest (POI) data, compared to local council food outlet data for the county of Cambridgeshire.

**Methods:**

Percentage agreement, positive predictive values (PPVs) and sensitivities were calculated for all food outlets across the study area, by outlet type, and across urban/rural/SES divisions.

**Results:**

Percentage agreement by outlet type (29.7-63.5%) differed significantly to overall percentage agreement (49%), differed significantly in rural areas (43%) compared to urban (52.8%), and by SES quintiles. POI data had an overall PPV of 74.9%, differing significantly for Convenience Stores (57.9%), Specialist Stores (68.3%), and Restaurants (82.6%). POI showed an overall ‘moderate’ sensitivity, although this varied significantly by outlet type. Whilst sensitivies by urban/rural/SES divides varied significantly from urban and least deprived reference categories, values remained ‘moderate’.

**Conclusions:**

Results suggest POI is a viable alternative to council data, particularly in terms of PPVs, which remain robust across urban/rural and SES divides. *Most* variation in completeness was by outlet type; lowest levels were for Convenience Stores, which are commonly cited as ‘obesogenic’.

## Background

Interest in the role food environments play in shaping behaviours related to food consumption and food choice has grown in recent years. Researchers have often studied this relationship between individuals and their environments through creating metrics of environmental ‘exposure’ [1], for example neighbourhood availability of fast food outlets [2-6]. However, the resulting evidence base is equivocal and the degree to which the environment determines behaviour remains unknown. In terms of study design, investigations into the ‘obesogenic environment’ [7] are frequently large scale, quantitative, often Geographical Information Systems (GIS) based [1,8-11], and importantly, rely heavily on the use of secondary data. Despite this, *relatively* little is known about the accuracy of commonly used secondary food environment datasets. In creating measures of food environment exposure that hope to *realistically* model individual-environment relationships, having accurate food outlet location data is critical, and so data accuracy should be better understood.

Several recent studies have addressed the accuracy and reliability of secondary food outlet data sources in relation to their utility for use in health research [12-20], although most assessments have been made in the US. Whilst collecting primary food outlet data might be the ideal, primary data collection is resource and time intensive. There is therefore an important place for secondary data in the quantification of food environments, yet the quality and completeness of such data are not always clear. In the US, companies such as Dun and Bradstreet (D&B) and InfoUSA can provide a minimal-fuss, geographically large and ready classified dataset, whilst in the UK, commercial Yellow Pages data can be purchased in bulk through providers such as Experian. The use of such data represents the lowest time resource cost option for secondary data acquisition. ‘Collecting’ data from local councils (governing bodies at the local level) or state departments is more complex, requiring a substantial time and resource investment to both obtain and streamline the data prior to use [21]. These three types of data source (‘primary’, ‘intensive secondary’ (such as council data), and ‘extensive secondary’ (such as Yellow Pages or InfoUSA data)) are all potentially important, allowing accuracy to be traded for convenience where imperatives such as research timelines prevail. However, in order to make the best decisions about which data to use, it is important to know how these different data sources compare.

Lake *et al.*[12] compared online and paper editions of the Yellow Pages telephone directories to the gold standard of a ground truthed food outlet database in North East England, finding positive predictive values (PPVs) of 79.1% and 82.4%, respectively. Even better was the PPV for food outlet data from local councils’ environmental health departments as compared with reality, at 91.5% in this area [12]. In the UK, food outlets are required to register their business with local councils by law in order to facilitate routine hygiene inspections, which may explain this accuracy. Other UK studies have re-iterated the accuracy of council records, reporting PPVs of 86.6% (1997) and 87.3% (2007) at two time points in Glasgow [13], and between 79-87% across urban/rural and socio-economic divides in North East England [14]. The sensitivity of council data compared to ‘reality’ has consistently shown itself to be ‘moderate’ to ‘excellent’ [14,15], according to a classification system developed by Paquet *et al.*[16]. In North America, although the accuracy of state level data was questioned in one paper [17], improved PPVs and sensitivities have been found for state level food records (ground truthed data as the gold standard) as compared with the much used D&B and InfoUSA commercial datasets [18-20].

This said, most assessments of data validity have been made across entire study areas, not accounting for differences in completeness across socio-economic lines or urban/rural boundaries. There is some suggestion that the accuracy of food outlet records may vary systematically across such divides [14,22], which do exist in the UK, albeit perhaps less overtly than in the US, for example. Whilst one small study in North East England did not find any significant differences in data validity by area SES or urban/rural status [14], potential differences in data integrity across these divides are important to consider as they might imbue systematic biases in downstream analyses.

In the UK, Ordnance Survey (OS) Points of Interest (POI) data are increasingly used in the literature as a source of information on environmental attributes such as the locations of food stores or physical activity facilities [23-25], and hold potential to be an accurate and useful source of ‘extensive secondary’ data due to its updateability, positional accuracy (co-ordinates are provided for environmental attributes with 1m precision), and theoretical comprehensiveness [26]; POI contains information from over 170 data suppliers, chosen for being “the most authoritative source…for the particular type of feature they supply and for the quality and completeness of [their] data” [26]. Inaccuracies demonstrated in other sources of commercial data only enhance the appeal of POI [12], however the accuracy of these data has not yet been assessed in the published academic literature, leaving its efficacy for use in health research in question.

Using accurate council food outlet location data as the reference standard, this study aims to assess the validity of POI data for use in research into the (obesogenic) food environment for the first time, in Cambridgeshire, UK. Reliability will be assessed as the completeness of POI records as compared to council data, which has been shown to be moderately to highly accurate in other regions of the UK, with a PPV of 91.5% in North East England [12]. We aim to undertake this assessment for all POI records across the study area and to assess whether POI completeness varies by outlet type, by urban/rural status and across socio-economic divides.

## Methods

### Food outlet data

**Figure 1:**
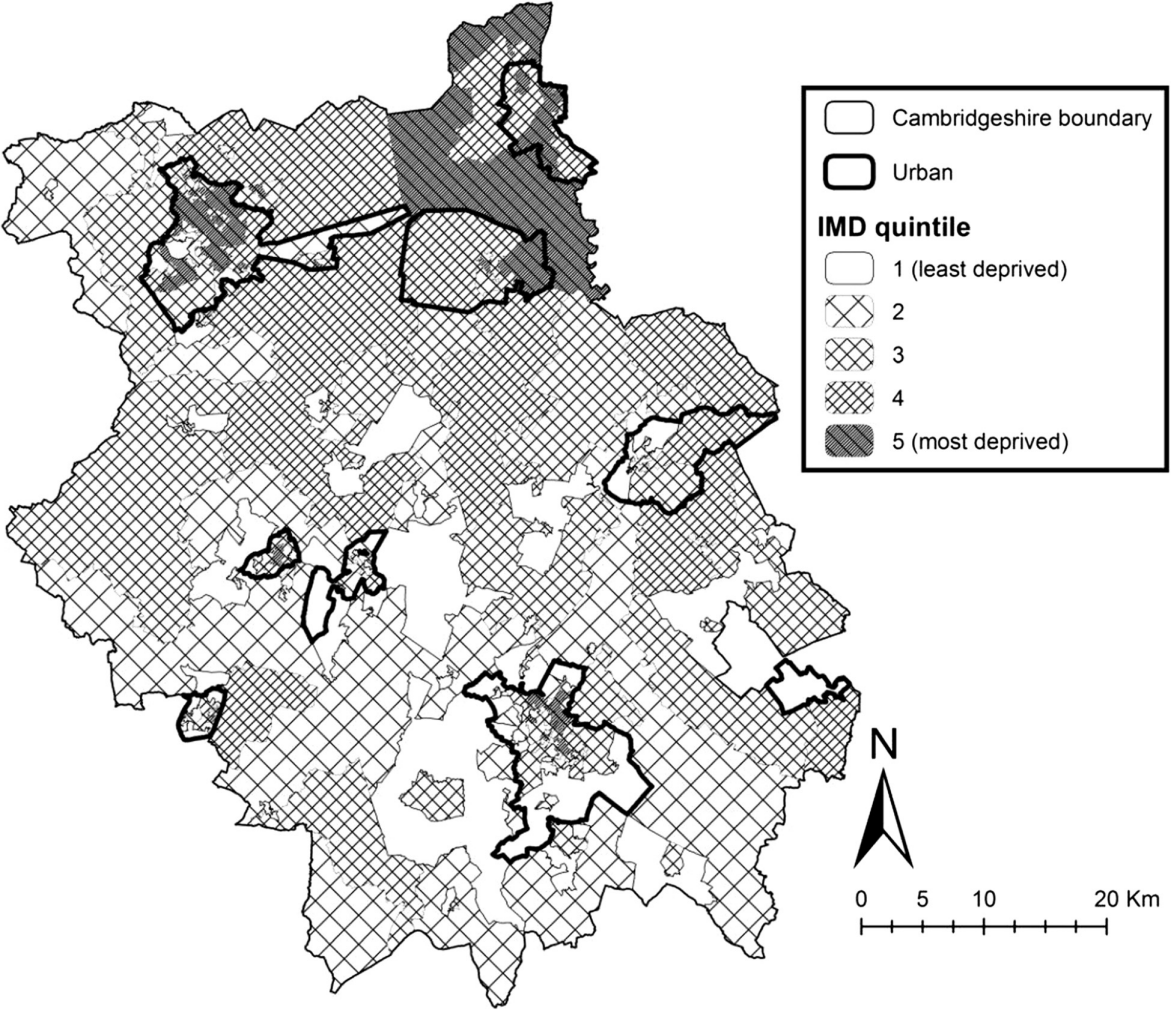
**Cambridgeshire county study area, showing Urban areas (based on lower super output area urban/rural classifications from Communities and Local Government) and Deprivation (Index of Multiple Deprivation) Quintiles.**^©^Crown Copyright/database right 2012. An Ordnance Survey/EDINA supplied service.

Data on the locations of food outlets throughout Cambridgeshire, UK (Figure 1), were sourced directly from OS under an educational license, and from local councils (n=6) throughout the region. Councils were approached individually and asked to provide their *current* environmental health food outlet records under the Freedom of Information (FOI) act (for details, see http://www.legislation.gov.uk/ukpga/2000/36/data.pdf). Both datasets were obtained in January 2012; minimising temporal mismatch between datasets was critical in making as fair a comparison as possible. Duplicate records (n=5) were identified in the council food outlet records received, and removed. Where no further address details were available, duplicate postcodes were assumed to represent co-existent food outlets, as postcodes usually contain multiple addresses. Food outlets from both council and POI datasets were classified according to a modified 6-point food outlet classification scheme, adapted from the 21-point schema developed by Lake *et al.*[12]. Any proprietary classification system already in place in the POI and council data received, was ignored. Each outlet was classified only once, according to its primary trading purpose, as has been done previously [12,14]. Food outlets were classified using internet research, Google Street View, phone calls, and local knowledge, by a single researcher to eliminate inter-rater bias, as either: ‘Café/Coffee Shops’, ‘Restaurants’, ‘Specialist Stores’ (butchers, ‘traditional’ bakers, fishmongers and so on), ‘Convenience’, ‘Supermarkets’ (defined as belonging to a major UK supermarket chain, such as Tesco, ASDA or Sainsbury’s and differentiated as such from independently owned traditional convenience stores) or ‘Takeaways’. These are broad categories of food outlet type, all potentially related to behaviours, as evidenced by the frequency of use of such categories in the published literature [27-33]. Public houses (‘pubs’) were considered individually and included as ‘Restaurants’ only if they sold food that was more than just ‘bar snacks’. Mobile food outlets were excluded from the datasets as the home address of the owner was often given in lieu of the retail location.

Outlets were matched based on their name, address and postcode. Outlets were matched, even where spelling of business name was similar but not identical, where supporting evidence (such as the same address and/or postcode) was present. Food outlet locations for council and POI data were geocoded according to their postcodes and overlaid atop Lower Super Output Area (LSOA) boundaries for Cambridgeshire, using ArcGIS 10 (ESRI Inc., Redlands, CA). LSOAs were attributed an urban/rural status (according to Communities and Local Government guidelines, defining small towns, villages and hamlets with fewer than 10,000 residents as ‘rural’ [34]), with a good mix of urban and rural areas present throughout the study area, as shown in Figure 1. LSOAs were also attributed a measure of area level socio-economic status (SES) (quintiles of Index of Multiple Deprivation (IMD) scores 2010 [35], relative to Cambridgeshire county), as also shown in Figure 1. IMD is a compound measure of SES across seven principle domains (income deprivation, employment deprivation, crime, health deprivation and disability, education, skills and training deprivation, barriers to housing and services and living environment deprivation), with scores increasing as deprivation increases [36].

### Statistical analyses

Completeness of POI data compared to the reference standard council data was assessed by calculating percentage agreement, positive predictive values (PPVs) and sensitivities for all outlets, and by type of food outlet, using PASW Statistics 18 (PASW Statistics Inc., Chicago, 2009). These statistics have been widely employed in the literature to date [12-14,16,18,19]. Percentage agreement computes the percentage of food outlets present in both POI and council data (true positives/(true positives + false negatives + false positives)). PPVs represent the percentage of outlets listed in the POI dataset that were also present in the council data (true positives/(true positives + false positives)). Sensitivity represents the percentage of outlets listed in the council data that were also listed in the POI data (true positives/(true positives + false negatives)). As is common in the literature, accepted sensitivity cut-offs will be applied here [16]: ‘poor’ <30%; ‘fair’ 31-50%; ‘moderate’ 51-70%; ‘good’ 71-90%; ‘excellent’ >91%. Lake *et al.*[12] present a useful diagram showing how PPVs and sensitivities are calculated and relate to each other. Differences between PPVs, sensitivities and percentage agreements for all food outlets as compared to food outlets by type were assessed using Fisher’s Exact tests (preferred over chi-squared tests due to potentially small expected values). PPVs and sensitivities were calculated separately for urban and rural areas and for each IMD quintile; comparisons with PPVs and sensitivities in relation to urban and least deprived reference categories were again made using Fisher’s Exact tests. A value of p<0.05 was used as the marker of statistical significance for differences.

## Results

### Percentage agreement

Descriptive statistics for council and POI data received are shown in Table 1. The POI data contains 524 fewer total records than were present in the council data, and fewer records by all types of food outlet, with the exception of supermarkets. For cafés/coffee shop records, POI data contained 39.07% fewer gross records. Table 1 also shows percentage agreement between council and POI data, across all food outlets, food outlets by type, and all food outlets across urban/rural divides and SES quintiles. Agreement varied according to food outlet type and was significantly different (p<0.05) to overall food outlet agreement (49.9%), with the exception of specialist food retailers. Percentage agreement was significantly lower in rural than urban reference areas (p<0.001). Compared to the least deprived reference areas, the third and fourth SES quintiles had significantly improved percentage agreement; other deprivation quintiles were not significantly different.

**Table 1:** Descriptive statistics and percentage agreement for all food outlets, food outlets by type, and all food outlets across urban/rural divides and socio-economic status quintiles

**Food outlet category**	**Council data**		**POI data**		**Missing POI records (%)**	**Percentage agreement (%)**^**a**^	**95% CI**	**95% CI for difference**
	**n**	**%**	**n**	**%**				
**All Food Outlets**	2624	100.00	2100	100.00	19.97	49.9 (*REF*)	0.482, 0.517	*REF*
Café/Coffee Shop	366	13.65	223	10.62	39.07	40.6**	0.358, 0.454	0.043, 0.144
Convenience	608	23.17	398	18.65	34.54	29.7**	0.265, 0.330	0.166, 0.239
Restaurant	852	32.47	757	36.05	11.15	63.5**	0.604, 0.665	−0.171, -0.101
Specialist Stores	248	9.45	221	10.52	10.89	47.5	0.419, 0.531	−0.033, 0.082
Supermarket	92	3.51	93	4.43	0.00	62.3*	0.527, 0.712	−0.214, -0.033
Takeaway	458	17.45	408	19.43	10.92	58.6**	0.543, 0.628	−0.132, -0.042
***Urban/Rural***								
Urban	1721	65.59	1484	70.67	13.77	52.8 (*REF*)	0.506, 0.549	*REF*
Rural	903	34.41	616	29.33	31.78	43.0**	0.400, 0.461	0.061, 0.134
***SES Quintiles***								
SES-1 (Least Deprived)	342	13.03	234	11.14	31.58	41.2 (*REF*)	0.364, 0.461	*REF*
SES-2	376	14.33	278	13.24	26.06	44.7	0.400, 0.494	−0.101, 0.031
SES-3	602	22.94	552	26.29	8.31	55.0**	0.513, 0.586	−0.198, -0.078
SES-4	627	23.89	523	24.90	16.59	53.9**	0.503, 0.576	−0.187, -0.068
SES-5 (Most Deprived)	677	25.80	513	24.43	24.22	45.1	0.417, 0.486	−0.098, 0.019

^a^ Significant difference (Fisher’s Exact, **p<0.001, *p<0.05) between food outlet category/area type and reference category (*REF*) within food outlet category/area type.

### Positive predictive value analysis

An ideal PPV would be 100%, whereby all outlets identified in the POI data were also present in the council data. Table 2 presents PPVs for all food outlets throughout the study area, and food outlets by type. The POI data has a PPV of 74.9% overall, with PPVs ranging between 57.9-82.6% by type. PPVs for Convenience and Specialist Stores and Restaurants were significantly different. PPVs across urban/rural areas and SES quintiles are also presented (Table 2), and are similar to urban and the least deprived quintile reference categories.

**Table 2:** PPVs for all food outlets, food outlets by type, and all food outlets across urban/rural divides and socio-economic status quintiles

**Food outlet category**	**PPV (%)**^**a**^	**95% CI**	**95% CI for difference**
**All Food Outlets**	74.9 (*REF*)	0.730, 0.768	*REF*
Café/Coffee Shop	76.2	0.701, 0.817	−0.072, 0.046
Convenience	57.9**	0.529, 0.628	0.118, 0.222
Restaurant	82.6**	0.797, 0.852	−0.109, -0.043
Specialist Stores	68.3*	0.618, 0.744	0.002, 0.130
Supermarket	76.3	0.664, 0.845	−0.102, 0.074
Takeaway	78.4	0.741, 0.823	−0.079, 0.009
***Urban/Rural***			
Urban	74.6 (*REF*)	0.723, 0.768	*REF*
Rural	74.2	0.705, 0.776	−0.037, 0.045
***SES Quintiles***			
SES-1 (Least Deprived)	71.8 (*REF*)	0.656, 0.775	*REF*
SES-2	72.7	0.670, 0.778	−0.087, 0.069
SES-3	74.2	0.704, 0.778	−0.093, 0.044
SES-4	77.1	0.732, 0.806	−0.121, 0.015
SES-5 (Most Deprived)	72.1	0.680, 0.760	−0.073, 0.066

^a^ Significant difference (Fisher’s Exact, **p<0.001, *p<0.05) between food outlet category/area type and reference category (*REF*) within food outlet category/area type.

### Sensitivity analysis

Results of sensitivity analyses are presented in Table 3, with Paquet *et al’s* sensitivity cut-offs applied [16]. Sensitivity for all food outlets throughout the study area was 59.9% (‘moderate’), and varied, mostly significantly, according to food outlet type (as high as 77.2% for supermarkets, p<0.05). Sensitivities were also both ‘moderate’ across urban/rural divides, although sensitivity in rural areas was significantly different to urban reference regions in terms of the sensitivity value proper. Although sensitivity in quintile 1 of SES is described as ‘fair’, it is borderline ‘moderate’, in line with other SES quintiles. This said, sensitivity values within SES quintiles 3 and 4 were significantly greater than in the most affluent reference category (p<0.001).

**Table 3:** Sensitivity values for all food outlets, food outlets by type, and all food outlets across urban/rural divides and socio-economic status quintiles

**Food outlet category**	**Sensitivity (%)**^**a**^	**95% CI**	**Sensitivity category **^**b**^	**95% CI for difference**
**All Food Outlets**	59.9 (*REF*)	0.580, 0.618	Moderate	*REF*
Café/Coffee Shop	46.4**	0.412, 0.517	Fair	0.081, 0.189
Convenience	37.8**	0.340, 0.418	Fair	0.178, 0.264
Restaurant	73.4**	0.703, 0.763	Good	−0.169, -0.099
Specialist Stores	60.9	0.545, 0.670	Moderate	−0.073, 0.054
Supermarket	77.2*	0.672, 0.853	Good	−0.260, -0.084
Takeaway	69.9**	0.654, 0.740	Moderate	−0.145, -0.053
***Urban/Rural***				
Urban	64.6 (*REF*)	0.621, 0.666	Moderate	*REF*
Rural	50.6**	0.473, 0.539	Moderate	0.098, 0.177
***SES Quintiles***				
SES-1 (Least Deprived)	49.1 (*REF*)	0.437, 0.546	Fair	*REF*
SES-2	53.7	0.485, 0.588	Moderate	−0.119, 0.027
SES-3	67.9**	0.640, 0.717	Moderate	−0.253, -0.123
SES-4	64.3**	0.604, 0.680	Moderate	−0.216, -0.087
SES-5 (Most Deprived)	54.7	0.508, 0.584	Moderate	−0.120, 0.010

^a^ Significant difference (Fisher’s Exact, **p<0.001, *p<0.05) between food outlet category/area type and reference category (*REF*) within food outlet category/area type.

^b^ Paquet *et al’s s*ensitivity category cut-offs: ‘poor’ <30%; ‘fair’ 31-50%; ‘moderate’ 51-70%; ‘good’ 71-90%; ‘excellent’ >91%.

## Discussion

This work examined the validity of a potentially important and increasingly used ‘extensive secondary’ dataset in the UK. As has been noted, despite general epidemiological concern with regards to measurement accuracy [18] and the determination of exposure ‘truth’ [37], surprisingly little is known about the validity of commonly used secondary data sources in the field. This study assessed the accuracy of POI data (at least as compared to previously validated local council records) for the first time in the published literature. Although the results of this study are therefore specific to POI data, as compared with local council records in Cambridgeshire, UK, the importance of considering the validity of secondary data in these ways and across pertinent divisions remains important across all secondary datasets; this study is novel in this respect.

In terms of concordance between the datasets, the POI data contained 524 fewer gross records than were present in the council data, with a percentage agreement of 49.9%, translating into an overall PPV of 74.9% and sensitivity of 59.9% (‘moderate’). These results are largely in line with previous studies examining the accuracy of *other* secondary food environment data [12-15,18-20], the caveat being that this study did not use a ground truthed dataset as a gold standard, and instead used a reliable secondary reference dataset (demonstrated to have a PPV of 91.5% in Newcastle, UK [12]) to increase the scale of the investigation.

Differentiation by type of food outlet revealed PPVs between 57.9% and 82.6%, with sensitivities between 37.8% (‘fair’) and 77.2% (‘good’). These assessments by food outlet type are roughly in line with those demonstrated in the literature [12,19], but rather below those shown for some commercial US datasets [18]. As these statistics were largely significantly sensitive to food outlet type, this research highlights the importance of considering the accuracy of secondary data for specific types of food outlet, as has been noted elsewhere [19]. Although we find the lowest levels of gross completeness for cafés/coffee shops (39%), in terms of the number of missing records in POI data, convenience store records are especially incomplete with regards to percentage agreement, PPVs and sensitivity. These small grocery shops are commonly cited as being ‘obesogenic’ [27,38,39], being less likely than larger supermarkets to sell ‘healthful’ foods [40]. Given this potential gap in the POI data, this might be an area to focus on if future research is considering supplementing POI data with either council records or field work. It is of note that POI appears to represent a particularly robust source of data on restaurant locations.

Importantly, PPVs across socio-economic and urban/rural divides were similar, both to each other, and to the statistic for all outlets. Such similarities have been demonstrated elsewhere [14,18]. For sensitivity and percentage agreement, there were exceptions, including significantly better estimates of both in some more deprived quintiles, although no evidence of a trend existed, and in urban areas. This said, sensitivies across urban/rural and SES divides mostly remained ‘moderate’ and as such aligned with the overall sensitivity description. Whilst the data should still be seen as ‘imperfect’ [13], some had suggested that *substantial* differences in food outlet representation across SES and urban/rural divides such as those tested here might prevail [14,22], and whilst this hypothesis should be further tested in validation studies of other datasets, we do not believe this was the case here.

The utility of POI data may be research specific, however, if selected as a source of food outlet location data, we suggest they should be used with confidence particularly with respect to data completeness over socio-economic divides, in urban areas, and where research focuses on restaurant, supermarket or takeaway locations.

Strengths of this study include the fair comparison of contemporaneous datasets, the application of a 6 category food outlet classification scheme whose outlet types should relate directly to future deductive research, and its large geographical scale, which enabled an assessment of over 2000 food outlets in each dataset. In particular, using established statistics (percentage agreement, PPVs and sensitivies) across urban/rural and socio-economic divides allowed an assessment of the likelihood of systematic geographical differences in completeness. To our knowledge, this is the first time that such an appraisal has been made in the published literature on a large scale.

There were several key limitations to this study. In order to enable the large study area, field work was not conducted, choosing instead to use local council data as our ‘gold (reference) standard’. Local council data have been shown accurate in several other regions of the UK, however they are unlikely to be complete, resulting in a potential lack of comparability with previous studies that can relate directly to the food environment *reality*. Despite this limitation, the strength of results found here suggest that if council data are indeed less complete than we might hope, or are systematically incomplete (for example, across socio-economic divides) they are at least aligned in these respects with POI records. In order to maximise heterogeneity in socio-economic status throughout the study area, quintiles of SES were calculated relative to the study area only. Increased sensitivity in detecting SES differences between LSOAs was useful for these analyses, however, our findings may not be applicable to the most deprived locales, which are substantially under-represented throughout Cambridgeshire (IMD scores are positively skewed towards being lower (less deprived); mean IMD for Cambridgeshire=15.51 (SD=11.44), range of possible IMD scores for England as a whole 0.53-87.80). This potential limitation may lead to a lesser degree of generalisability outside this study area, however it does not compromise the accuracy of *these* results. To facilitate a fair comparison of the datasets, we attempted to obtain as contemporaneous information as possible. We asked OS and local councils for current data in January 2012 to facilitate this, however, it is possible that either dataset may not reflect the food environment at precisely the same time. Whilst some exclusions in the datasets were made based on food not sold directly to the public (food producers, for example), exclusions of market traders or mobile food stands were made predominantly because addresses were for the traders’ home addresses and not the retail sites themselves. These types of food retailers are likely important sources of food [14,22], potentially with a socio-economic gradient of use [41,42], and should be considered where possible in future validation work.

In terms of the POI dataset itself, the data were not without duplicates that needed to be found and removed (n=105). The classification system supplied was too general to be of real use in most health research (for details see, http://www.ordnancesurvey.co.uk/oswebsite/docs/product-schemas/points-of-interest-classifications-scheme.pdf) so a project specific classification scheme such as the one used here would almost certainly be required. POI contains records beyond simply the foodscape, making it difficult to discern whether listed establishments sold food or not. In council datasets, outlets are listed *precisely because* they sell food. This breadth may lead to the omission of important sources of food within the environment, for example from pharmacies, such as Boots the Chemist, a national chain that often but not always sells food items. Investigative work would be required when using POI data to determine whether or not each of these individual stores sells food.

## Conclusions

Accurate analysis in health and policy research begins with accurate data. Ordnance Survey Points of Interest records generally compared favourably here in relation to data from local councils’ environmental health departments. We observed few notable systematic variations in POI completeness (PPV/sensitivity) over urban/rural and SES divides, however when type of outlet was considered, convenience stores appeared to be the least well represented in the POI, and consideration must therefore be given to the types of outlets being studied when selecting a dataset.

The utility of POI is boosted when its relative ease of acquisition is considered (in relation to both ‘intensive secondary’ council data, and primary data collection). However, this is not to say that by *combining* POI data with local council data, one might be able to build an even more accurate picture of the food environment. Future research using a ground truthed dataset over an equivalent study area is necessary to ascertain whether this is likely to be the case.

## Competing interests

The authors declare they have no completing interests.

## Authors’ contributions

The study design was jointly devised by TB and FH. TB was responsible for data collection from local councils, FH for data acquisition from Ordnance Survey. TB led on data analysis. TB and FH drafted the manuscript together. Both authors read and approved the final manuscript.
